# Risk factors and treatment advances for carbapenem-resistant Enterobacteriaceae infections in the urinary tract

**DOI:** 10.3389/fcimb.2025.1724851

**Published:** 2026-01-08

**Authors:** Xuming Zhang, Shouyu Miao, Chaoyue Ji, Weiguo Hu

**Affiliations:** 1 Department of Urology, Beijing Tsinghua Changgung Hospital, School of Clinical Medicine, Tsinghua Medicine, Tsinghua University, Beijing, China; 2 Department of Rheumatology, The First Hospital of Jilin University, Changchun, China

**Keywords:** carbapenem-resistant Enterobacteriaceae, cefiderocol, risk factors, treatment, urinary tract infections

## Abstract

Carbapenem-resistant Enterobacteriaceae (CRE) pose a significant threat in urinary tract infections (UTIs), which are among the most common infectious diseases. This review summarizes the risk factors and treatment advances for CRE-associated UTIs. Key risk factors include advanced age, history of carbapenem use, invasive urological procedures, indwelling catheters, immunosuppression (e.g., in transplant recipients), and prolonged hospitalization. The prognosis of CRE UTIs is generally better than infections at other sites but is influenced by factors like ICU admission and treatment failure. Treatment relies heavily on novel antibiotics, such as ceftazidime-avibactam and the siderophore cephalosporin cefiderocol, often used in combination regimens to enhance efficacy and prevent resistance. Recent clinical trials have demonstrated the effectiveness and safety of cefiderocol against CRE UTIs. Research on adjuvants like metal ion chelators (e.g., copper, bismuth) to restore antibiotic susceptibility is promising. Future efforts should focus on developing new antimicrobials, establishing rapid diagnostics for precise control, and conducting large-scale studies to optimize individual treatment strategies and infection control measures to improve patient outcomes and curb CRE spread.

## 1 Introduction

The term ‘carbapenem-resistant Enterobacteriaceae’ (CRE) is used to denote a particular category of Enterobacteriaceae, as defined by the Centers for Disease Control and Prevention in the United States. These bacteria are characterized by their resistance to at least one carbapenem antibiotic, or by their capacity to produce carbapenemase (Bologna et al., 2024). In accordance with the regulations stipulated by the Clinical and Laboratory Standards Institute of the United States with regard to the minimum inhibitory concentration (MIC) in drug susceptibility tests, the MIC of doripenem, meropenem and imipenem against CRE is ≥ 4 µg/mL, and the MIC of ertapenem against CRE is ≥ 2 µg/mL (Potter et al., 2016). Urinary tract infections (UTIs) are among the most prevalent infectious diseases in medical settings. Annually, approximately 150 million individuals worldwide are impacted by this condition (Tandogdu and Wagenlehner, 2016). The majority of UTIs are caused by Enterobacteriaceae bacteria, thus making the urinary system a significant site for the occurrence of CRE. However, there is a paucity of literature focusing on CRE-associated UTIs. The present article aims to provide a comprehensive introduction to the epidemiology and virulence of CRE, as well as a systematic review of the risk factors and treatment progress of CRE-associated UTIs. The expectation is that this will assist urologists in acquiring a more profound understanding of CRE and facilitate their clinical practice.

## 2 The epidemiology and hazards of CRE

In 2017, the World Health Organization (WHO) published a list of three types of drug-resistant bacteria that most urgently require the development of new antibiotics on a global scale (Tacconelli et al., 2018). The most prevalent bacterial types are carbapenem-resistant *Klebsiella pneumoniae* (CRKP) and carbapenem-resistant *Escherichia coli* (CREco). Furthermore, Citrobacter has been identified as the third most common bacterial species. According to the latest data from the WHO, the median resistance rate of Klebsiella pneumoniae isolated from the urinary tract to different types of carbapenem antibiotics ranges from 5% to 15% in all countries, while this rate for Escherichia coli is between 1% and 5%. According to data from China (China Antimicrobial Surveillance Network, 2024), the world’s largest developing country, the resistance rate of *Klebsiella pneumoniae* to carbapenem antibiotics exceeds 20%, and that of *Escherichia coli* exceeds 2% in 2024. This finding indicates that the prevalence of CRE resistance in developing countries exceeds the international average. In terms of population characteristics, a study involving 25 tertiary hospitals indicated that the median age of patients with CRE infection was 62 years old (Zhang et al., 2018). Evidence suggests that the detection rate is higher in areas with a larger elderly population, and that more active prevention and control measures for CRE should be taken. The deleterious nature of CRE is indisputable. A comprehensive meta-analysis article to date has concluded that the mortality rate of patients infected with CRE is significantly higher than that of patients infected with carbapenem-sensitive Enterobacteriaceae, and in some studies, it can even be more than twice as high (Zhou et al., 2021).

The prognoses of CRE infection vary according to the affected anatomical region. Among them, the prognosis of CRE infection in the nervous system is the worst. In comparison with patients infected with CRE in other parts of the body, patients with CRE urinary tract infection have a relatively better prognosis. Shilo et al (Shilo et al., 2013). Demonstrated that there was no difference in in-hospital mortality between patients with CRKP and those with carbapenem-sensitive *Klebsiella pneumoniae* (CSKP) urinary tract infections. Subsequently, Qureshi et al (Qureshi et al., 2014). the majority of patients with CRE bacteriuria were found to be asymptomatic, and no fatalities due to infection were observed. Among those exhibiting symptoms, over 90% experienced a resolution of symptoms following treatment with systemic anti-infective medications. Carrilho et al (de Maio Carrilho et al., 2016). found that urinary tract was a protective factor for death caused by CRE infection among all sites. This finding supported the conclusion of the above-mentioned study. At present, the factors influencing the prognosis of CRE patients with UTIs that are known include ICU admission, failure of antibiotics to eradicate bacteria, and history of ertapenem use (Önal et al., 2020), etc. In addition, research conducted by Pouch (Pouch et al., 2015) et al. and Varotti (Varotti et al., 2017) et al. has demonstrated that patients who have undergone kidney transplantation exhibit an elevated mortality rate following postoperative infection with CRKP and are more susceptible to the loss of the transplanted organ. Bratosin et al (Bratosin et al., 2024). revealed the complex interaction between microbial resistance and pregnancy outcomes. The study compared the types of pathogenic bacteria causing UTIs during pregnancy between preterm and full-term pregnant women and found that the proportion of CRE was higher in preterm pregnant women. In conclusion, the clinical outcomes of patients with CRE-associated UTIs are significantly different due to their different clinical characteristics. It is therefore vital that clinicians fully consider the particularity of these patients in clinical practice. At the same time, given the paucity of studies on the prognostic factors of CRE-associated UTIs, large-sample prospective studies are still needed in this field to clarify and guide clinical practice.

## 3 The risk factors for CRE-associated UTIs

UTIs are among the most extensively studied infection types associated with CRE worldwide at present. Nevertheless, the majority of research on CRE-associated UTIs concentrated on drug treatment, with relatively few studies addressing risk factors. The earliest study, dating back to 2012, indicated that a high number of antibiotics used, indwelling urinary catheters, previous surgical history, invasive procedures such as catheterization and endoscopy, and ICU admission might be risk factors for CRKP UTIs (Shilo et al., 2013). However, the presence of patients with dementia in the study population may have introduced a recall bias, thereby compromising the study’s reliability. In the ensuing study, ÖNAL et al. initially delineated the clinical manifestations exhibited by patients following a CRE urinary tract infection, which exhibited a higher propensity to manifest in the upper urinary tract. The prevailing symptoms encompassed fever, dysuria, and discomfort in the abdominal or groin region (Önal et al., 2020). At the same time, over 70% of the patients in this study had undergone invasive procedures or surgeries, which might be an important cause of CRE infection. Kizilay (Kizilay et al., 2020) et al. conducted a study that distinguished the infection risks associated with different types of catheters in the urology field. Their findings indicated that a higher rate of nephrostomy tube retention upon admission could be a significant risk factor for CRKP infection via the extraluminal route. However, no statistical difference was observed in the retention rates of other types of catheters. The study did not specify the point in time at which CRKP was detected. Given that the study also recorded the treatment process of each patient after admission, it can be posited that the patients with CRE-associated UTIs included in this study were those throughout the entire hospital stay. Therefore, it can be concluded that there is a certain limitation in the early prediction upon admission.

In the context of research conducted on specific populations, the findings of Forster (Forster et al., 2017) et al. suggested that children who undergo long-term intermittent catheterization exhibit a considerably elevated prevalence of CRE infection. This phenomenon might be associated with prolonged antibiotic utilization and the existence of neurogenic bladder. Kim (Kim et al., 2024) et al. demonstrated that stroke patients with hypertension are more prone to CRE-associated UTIs, which may be related to the difficulty of maintaining hygiene for stroke patients and the damage to the immune system caused by hypertension. In the domain of kidney transplantation, there exists a plethora of studies addressing CRE-associated UTIs. This is attributable to the fact that UTIs are more prevalent among transplant patients than in the non-transplant population, and they constitute the most prevalent type of infection following kidney transplantation (Brizendine et al., 2015). Patients who have received transplants have more risk factors for infection with drug-resistant bacteria, including long-term hospitalization, admission to the ICU, undergoing invasive procedures, hemodialysis and immunosuppression, etc. Freire (Freire et al., 2020) et al.’s research found that 14.6% of the pathogenic bacteria causing UTIs symptoms after kidney transplantation were CRE. UTIs caused by CRE or surgical site infections in patients who have undergone kidney transplantation usually occur within one month after the operation (Rodrigues Dos Santos et al., 2016). This is prior to the period in which infections caused by sensitive bacteria occur, and the proportion of cases that progress to bacteremia is higher (Pouch et al., 2015).

The independent risk factors for postoperative urinary tract infection with CRE in kidney transplant recipients that have been identified to date include diabetes, a longer hospital stay and recent use of antibiotics other than trimethoprim-sulfamethoxazole (TMP-SMX). Another study identified some possible risk factors for urinary tract infection with CRKP in kidney transplant recipients, including a lower body mass index (BMI) and a higher Clavien-Dindo score (Varotti et al., 2017). The relationship between BMI and the nutritional status of patients has been well documented. In cases of malnutrition, there is a limited proliferation of immune cells, reduced protein synthesis, and an elevated risk of infection. The Clavien-Dindo classification score is a widely accepted surgical complication classification score. High-severity surgical complications have been shown to be associated with prolonged hospital stays and the use of high-level antibiotics. The recurrence rate of UTI caused by CRE is very high. The study by Freire (Freire et al., 2020) et al. also investigated the risk factors for recurrent UTIs after kidney transplantation. The study proved that diabetic nephropathy leading to end-stage renal disease, UTIs within 180 days after transplantation, anatomical changes at the time of UTIs diagnosis, and effective treatment time are its risk factors. The article points out that the effective treatment time is a very controversial issue because a shorter time significantly increases the incidence of recurrent UTIs, while a longer time reduces the treatment compliance of patients. Therefore, a standard treatment time still requires a large amount of research.

It is important to note that there is another study on the risk factors for CRE infection in postoperative renal transplant patients that is relevant for review here. However, this study did not distinguish the sites of infection that occurred postoperatively. Freire (Freire et al., 2021) et al. conducted a large case-control study involving nearly 1,000 patients who had undergone kidney transplantation. This study identified factors associated with postoperative CRE infection, including older age, diabetic nephropathy as the cause of end-stage renal disease, delayed graft function recovery, placing ureteral stents, lower median lymphocyte count, and acute cellular rejection. The conclusion drawn from this study was not entirely consistent with previous research on CRE in urinary tract infections. In cases of poorly accepted organs and acute cellular rejection, increased levels of immunosuppressive drugs are typically required, along with prolonged hospitalization and a higher incidence of delayed graft function. Furthermore, a lower median lymphocyte count is indicative of a diminished immune response in patients. Whether to place a ureteral stent during transplantation is a controversial issue. The etiology of ureteral obstruction after renal transplantation encompasses a range of potential factors, including ureteral stenosis, peripheral hematoma, cyst-induced compression, and the presence of stones, among others. Among these etiologies, ureteral stenosis has been documented as the most prevalent, exhibiting an incidence rate of approximately 3% (Dinckan et al., 2007). The efficacy of ureteral stent placement in the management of ureteral obstruction after kidney transplantation has been substantiated by numerous studies (Xu et al., 2015; Sedigh et al., 2020; Spagnoletti et al., 2023). Ureteral stents effectively drain urine, preventing urinary stasis due to complications and significantly reducing the incidence of UTIs (Imvrios et al., 2019). However, the presence of an invasive device within the urinary tract has been demonstrated to increase the risk of biofilm formation and reduce bacterial clearance (Flemming et al., 2016). Therefore, ureteral stents may also become a breeding ground for bacterial colonization. As super-bacteria, CRE naturally tends to colonize the ureter stents, thereby inducing UTIs. Balancing the optimal utilization of ureteral stents to promote drainage against the risk of UTIs, Imvrios (Imvrios et al., 2019) et al. offered valuable insights. Their research indicated that prompt catheter removal within one month post-surgery, coupled with prophylactic antibiotic use during stent placement, significantly reduced the incidence of UTIs. This reduction was even lower than in patients who did not receive ureteral stents post-operatively.

As demonstrated in this study, the risk factors for CRE-associated UTIs are predominantly concentrated in advanced age, a history of carbapenem antibiotic use, invasive procedures, and a weakened immune system due to various factors (**Table 1**). Given that the majority of CRE isolated from urine cultures are hospital-acquired rather than community-acquired, future research directions should include the active prevention and management of CRE infections in high-risk patients following admission.

**Table 1:** Summary of key risk factors for CRE-associated UTIs.

Risk factor	Type classification	References (Document Citation Numbers)
Advanced age	Demographic factor	(China Antimicrobial Surveillance Network, 2024; Freire et al., 2021),
History of carbapenem use	Drug-related	(Qureshi et al., 2014; Tandogdu and Wagenlehner, 2016),
Invasive urological procedures (catheterization, endoscopy, etc.)	Invasive procedure/Device	(de Maio Carrilho et al., 2016; Rodrigues Dos Santos et al., 2016; Kizilay et al., 2020; Freire et al., 2021; Bratosin et al., 2024)
Immunosuppression (kidney transplant recipients)	Underlying disease/Immune status	(Önal et al., 2020; Freire et al., 2021; Kim et al., 2024)
Diabetes mellitus	Underlying disease/Immune status	(Freire et al., 2020; Freire et al., 2021)
Diabetic nephropathy-induced end-stage renal disease	Underlying disease/Immune status	(Brizendine et al., 2015; Freire et al., 2021),
Stroke with hypertension	Underlying disease/Immune status	(Forster et al., 2017)
Neurogenic bladder	Underlying disease/Immune status	(Kizilay et al., 2020)
Delayed graft function recovery	Healthcare-related factor	(Freire et al., 2021)
Prolonged hospitalization	Healthcare-related factor	(Freire et al., 2020; Freire et al., 2021),
ICU admission	Healthcare-related factor	(Tandogdu and Wagenlehner, 2016; Bratosin et al., 2024),
Higher Clavien-Dindo score	Healthcare-related factor	(Freire et al., 2020)
Anatomical changes in UTI diagnosis	Other	(Brizendine et al., 2015)

## 4 The treatment advances for CRE-associated UTIs

The most prevalent and significant treatment for CRE is the administration of antibiotics. The Food and Drug Administration of the United States (FDA) has approved a variety of drugs for the treatment of CRE infections in recent years. The 2024 guidelines for the diagnosis and treatment of multidrug-resistant bacteria, issued by the Infectious Diseases Society of America, indicate that the targeted drugs currently available for the clinical treatment of CRE mainly include meropenem-vaborbactam, ceftazidime-avibactam, imipenem-cilastatin/relebactam, amikacin, cefiderocol, colistin, tetracyclines, fosfomycin, and tigecycline, etc (Tamma et al., 2024). We compared the relevant drugs, as shown in **Table 2**. Clinically, appropriate treatment plans can be selected based on the type of infection, the severity of the infection, and the differences in the infecting strains. It is important to acknowledge that the aforementioned pharmaceuticals have not been introduced in all countries. Consequently, the selection of therapeutic drugs should be informed by the specific circumstances of each nation. With the exception of ceftazidime-avibactam when administered in isolation, the efficacy of other pharmaceuticals is enhanced when utilized in conjunction with one another. This phenomenon can be attributed to the ability of multi-drug combinations to impede the emergence of drug-resistant bacteria and expedite the management of infections. Furthermore, the implementation of multi-drug combinations enables a reduction in the dosage of highly toxic medications, thereby minimizing the occurrence of adverse drug reactions.

**Table 2:** Comparison of antimicrobial agents for CRE-associated UTIs.

Antimicrobial agent	CLSI susceptible breakpoint	Renal dosing for normal renal and hepatic function	Adverse effects
Cefiderocol	≤4 μg/mL	2 grams IV every 8 h, infused over 3 h	Nephrotoxicity, elevated liver enzymes, hypokalemia
Meropenem-Vaborbactam	≤1 μg/mL	1 grams IV every 8 h, infused over 30 min	Nausea/vomiting, headache, infusion-site reactions
Ceftazidime-Avibactam	≤2 μg/mL	2.5 grams IV every 8 h, infused over 3 h	Diarrhea, headache, fatigue
Imipenem-Cilastatin/Relebactam	≤1 μg/mL	1.25 grams IV every 6 h, infused over 30 min	Nausea, vomiting, abdominal pain
Colistin	–	5 mg/kg IV loading dose, then 2.5 mg/kg q12h	Nephrotoxicity, neurotoxicity
Fosfomycin	≤64 μg/mL	3 grams as a single dose	Diarrhea, nausea, abdominal cramps

The majority of contemporary FDA approvals, or those impending, pertain to cephalosporin antibiotics or aminoglycoside antibiotics in conjunction with a beta-lactamase inhibitor, such as aztreonam-avibactam and meropenem-vaborbactam. Nevertheless, there has been no breakthrough in terms of pharmacological action, with some β-lactamase enzymes exhibiting an absence of inhibitory activity against metal enzymes (Bologna et al., 2024). Therefore, this section of the paper focuses on some drugs that possess novel antimicrobial mechanisms. Cefiderocol is a novel cephalosporin with a catechol group and an iron carrier. It was approved by the FDA in 2019 for the treatment of complicated UTIs (Lee and Yeo, 2020). The distinctive antibacterial mechanism of cefiderocol is attributable to the chlorocatechol structure located on the C3 side chain of its molecular structure. This structural element is capable of mimicking the iron carrier that is secreted by bacteria. After binding with iron ions, the cefiderocol molecule can penetrate the bacterial outer membrane in a “Trojan horse” manner and eventually combine with penicillin-binding proteins, thereby killing the bacteria (Syed, 2021). The so-called “siderophore” is one of the most powerful iron-chelating molecules. Due to the scarcity of free iron in the human body, almost all microorganisms have evolved siderophores that can be secreted outside the cell (especially in some Gram-negative bacteria), thus facilitating the uptake of iron from iron-deficient environments (Abdul-Mutakabbir et al., 2020). The emergence of cefiderocol indicates that the combination of iron carriers and antibiotics is a new therapeutic approach. This drug is also recommended by the latest guidelines of the Infectious Diseases Society of America for complicated CRE-associated UTIs. Cefiderocol has a broad antibacterial spectrum and is effective against most Gram-negative bacteria, such as Enterobacteriaceae, Acinetobacter baumannii, and Pseudomonas aeruginosa (Gijón Cordero et al., 2022). To date, the two most valuable clinical trials of cefiderocol in the field of UTIs are the “APEKS-cUTI” trial and the “CREDIBLE-CR” trial. The “APEKS-cUTI” trial was a double-blind, randomized, non-inferiority study and was one of the main bases for the drug’s approval for the treatment of complicated UTIs (Portsmouth et al., 2018). This study included a total of 452 patients from around the world and was divided into groups at a ratio of 2:1, with cefiderocol and imipenem-cilastatin being administered respectively. The primary endpoints for efficacy were the clinical effect (whether symptoms improved, were relieved, or new symptoms emerged) and microbiological effect (whether the microbial count in urine culture was ≤ 1×10^4^ colony-forming units per milliliter, CFU/mL) on the 7th day of use. Safety was evaluated through the incidence of adverse events, clinical laboratory tests, and electrocardiograms. The final results showed that the clinical and microbiological effects of cefiderocol were not inferior to those of high-dose imipenem-cilastatin. The clinical cure rates were found to be similar between the two groups, and the microbial eradication rate was found to be significantly higher in the cefiderocol group. Adverse events in the cefiderocol group were mostly concentrated in gastrointestinal disorders such as diarrhea and constipation, and the incidence of both adverse and serious adverse events was found to be lower in comparison to the imipenem-cilastatin group. This study demonstrated that cefiderocol is an effective drug for treating complicated UTIs caused by Gram-negative bacilli, with good safety. However, the study included relatively few patients infected with multi-drug resistant bacteria, especially those infected with CRE. The subsequent “CREDIBLE-CR” trial addressed this limitation.

The “CREDIBLE-CR” trial mainly verified the efficacy of cefiderocol in treating infections caused by carbapenem-resistant Gram-negative bacilli (Bassetti et al., 2021). The present trial encompassed a diverse cohort of patients afflicted with various types of infections, including hospital-acquired pneumonia, bloodstream infections, and UTIs. These patients were methodically divided into two groups: the cefiderocol group and the “best treatment” group. The latter group comprised patients for whom the most appropriate treatment was selected on the basis of various factors, including geographical location, the nature of the condition, and other relevant criteria. The final results demonstrated that for patients with CRE (including metallo-β-lactamase-producing microorganisms) infections, the overall clinical cure rate and microbial eradication rate of cefiderocol were higher than those of the best treatment. This was also the case in the subgroup of complicated UTIs. Regarding safety, the all-cause mortality rate in the cefiderocol group was significantly higher than that in the control group, with the difference primarily concentrated in the *Acinetobacter baumannii* infection subgroup. Within this subgroup, patients in the cefiderocol group presented with more severe baseline conditions. Additionally, the cefiderocol group exhibited a markedly higher incidence of liver-related treatment-related adverse events compared to the control group. This suggests that when administering cefiderocol to patients with hepatic impairment or underlying liver disease, regular monitoring of liver function parameters is warranted, with dose adjustment as necessary. Current research on cefiderocol’s efficacy and safety remains limited, with no studies conducted in special populations such as children or pregnant women. Future high-quality, large-scale randomized controlled trials are required to further validate these findings.

In addition to the above-mentioned drugs, research on how to enhance the sensitivity and effectiveness of existing drugs is also one of the directions in the fight against multi-drug resistant bacteria in recent years. Although these novel therapies have not yet been translated into drugs for clinical use, their mechanisms warrant review here, as they hold promise for inspiring future drug research. Current studies have shown that copper ions can inhibit the activity of NDM-1 carbapenemase, and copper ion coordination complexes can enhance the sensitivity of NDM-1 positive Escherichia coli to carbapenem antibiotics. This may be because copper has a relatively higher affinity for the metal ion binding sites of these metal enzymes, which leads to the exchange of metal ions and thus disrupts the active sites of the metal enzymes (Djoko et al., 2018). There are also studies indicating that copper ions can inhibit the β-lactam drug resistance mediated by peptidoglycan LD-transpeptidase (Peters et al., 2018). The extant research suggests that copper ions may have great potential as adjuvants for β-lactam drugs (including carbapenem drugs). Besides copper ions, bismuth agents commonly used in the treatment of gastrointestinal diseases have been proven to competitively bind to the active center of Tet(X4) protein, while bismuth atoms non-competitively alter the structure of Tet(X4) protein (Deng et al., 2022). Both of these mechanisms antagonize the enzymatic activity of Tet(X4) resistance protein against tigecycline, thus bismuth agents can effectively enhance the antibacterial activity of tigecycline against tet(X4) gene-positive bacteria. In summary, there are still severe challenges and unknown areas in the antibiotic treatment plan for CRE-associated UTIs, and there is still a broad space to explore for the research and development of new drugs and adjuvants.

## 5 Conclusion

The management of CRE-associated UTIs requires a shift from broad protocols to actionable, patient-specific strategies. For clinicians, this translates to: 1) proactively identifying high-risk patients (e.g., post-transplant, with indwelling devices) for early screening; 2) utilizing rapid diagnostics when available to guide initial, targeted therapy, moving beyond empirical carbapenem use; and 3) tailoring treatment by carefully integrating drug-specific factors (e.g., cefiderocol’s hepatic metabolism, colistin’s nephrotoxicity) with patient variables such as renal function and comorbidities. For complex cases, combination regimens should be considered to improve efficacy and hinder resistance. Ultimately, integrating precise risk assessment, timely diagnostics, and pharmacologically-informed regimen selection is key to optimizing outcomes and curbing the spread of CRE.
